# On the Response of a Micro Non-Destructive Testing X-ray Detector

**DOI:** 10.3390/ma14040888

**Published:** 2021-02-13

**Authors:** Dionysios Linardatos, Vaia Koukou, Niki Martini, Anastasios Konstantinidis, Athanasios Bakas, George Fountos, Ioannis Valais, Christos Michail

**Affiliations:** 1 Radiation Physics, Materials Technology and Biomedical Imaging Laboratory, Department of Biomedical Engineering, University of West Attica, Ag. Spyridonos, 12210 Athens, Greece; dlinardatos@uniwa.gr (D.L.); koukou@uniwa.gr (V.K.); mmartini@uniwa.gr (N.M.); gfoun@uniwa.gr (G.F.); cmichail@uniwa.gr (C.M.); 2 Radiological Sciences Group, Department of Medical Physics, Queen Alexandra Hospital, Portsmouth Hospitals University NHS Trust, Portsmouth PO6 3LY, UK; Anastasios.Konstantinidis@porthosp.nhs.uk; 3 Department of Biomedical Sciences, University of West Attica, Ag. Spyridonos, 12210 Athens, Greece; abakas@uniwa.gr

**Keywords:** CMOS, imaging, Gd_2_O_2_S:Tb, ZnSe:Te, non-destructive testing, DQE, scintillators, IEC 62220-1-1:2015

## Abstract

Certain imaging performance metrics are examined for a state-of-the-art 20 μm pixel pitch CMOS sensor (RadEye HR), coupled to a Gd_2_O_2_S:Tb scintillator screen. The signal transfer property (STP), the modulation transfer function (MTF), the normalized noise power spectrum (NNPS) and the detective quantum efficiency (DQE) were estimated according to the IEC 62220-1-1:2015 standard. The detector exhibits excellent linearity (coefficient of determination of the STP linear regression fit, R^2^ was 0.9978), while its DQE peaks at 33% and reaches 10% at a spatial frequency of 3 cycles/mm, for the measured with a Piranha RTI dosimeter (coefficient of variation CV = 0.03%) exposure value of 28.1 μGy DAK (detector Air Kerma). The resolution capabilities of the X-ray detector under investigation were compared to other commercial CMOS sensors, and were found in every case higher, except from the previous RadEye HR model (CMOS—Gd_2_O_2_S:Tb screen pair with 22.5 μm pixel pitch) version which had slightly better MTF. The present digital imager is designed for industrial inspection applications, nonetheless its applicability to medical imaging, as well as dual-energy is considered and certain approaches are discussed in this respect.

## 1. Introduction

The last decades have witnessed an unprecedented growth in the development of X-ray digital imaging technologies, to the extent that it is yet difficult to imagine the modern radiology in their absence. Digital imagers have revolutionized the otherwise classic procedures such as radiography, but more importantly have provided advanced techniques like digital breast tomosynthesis (DBT) [1,2,3], digital subtraction angiography (DSA) [4], thin tissue autoradiography [5,6], X-ray phase contrast imaging (XPCi) [7], etc. In the industrial field, this technology finds application in non-destructive testing (NDT) as well. In this quality control method, real-time radiography (RTR) is used for projection imaging and inspection of components from a production line. Material characterization and evaluation of substance properties are further applications under the term non-invasive inspection techniques. X-ray refraction, for instance, utilizes digital imagers for the characterization of well-defined geometry samples (fibers, capillaries) or bulks of micro-particles [8,9,10].

Digital imagers in their indirect-detection version, i.e., coupled to a scintillator material, have turned up in various forms; amorphous silicon on thin-film-transistor panels (a-Si-TFT) and their high-purity counterparts, charge-coupled devices (CCD) and complementary metal-oxide semiconductors (CMOS), either active or passive pixel sensor (APS or PPS) [11,12].

CCD detectors exhibit high resolution, good response linearity, low dark signal, low read noise and high sensitivity, making them superior at low exposures [12,13]. Initially their active surface used to be limited to less than 7 cm^2^ [14], but today’s designs have reached 85 cm^2^ [15], albeit at a high cost. Those small-sized CCDs necessitate demagnification solutions for applications that require larger fields of view [16]. This inserts an additional source of noise and degrades image quality, up to the point of determining the whole system’s performance (quantum sink) [11,17,18]. Something which, along with the specialized manufacturing process that these devices demand, adds to the complexity and gives rise to the overall cost. Moreover, CCDs are prone to radiation damage [19,20].

CMOS imagers take advantage of their sharing a highly developed fabrication process with the semiconductor industry, a field that progresses in a frantic pace and continuously curtails costs. Hence, they are presently a cost-effective solution that offers very high frame rates, X-Y addressability and low power consumption [21]. Their detecting area can reach more than 160 cm^2^, while they can be designed 2–3 sides buttable [2,14], allowing placement in a tiled fashion and the building of a large area imager. The need for the (detrimental for the image quality) demagnification is eliminated and a direct coupling of the scintillator material is thus permitted. Regarding the attainable resolution, sub – 2 μm pixel sizes have been reported [22,23].

Initially, CMOS’s main disadvantage over CCD was the higher electronic noise [24]. Today’s APS designs, by integrating advanced functions like buffering and amplification on-chip and in-pixel, outperform in this respect. At the same time, they can support much higher read-out speeds because of their massively parallel architecture [7,25], that can be obtained, along with lower bandwidth–lower noise ADCs (analog to digital converters). Frame rates above 600 fps (frames per second) have been achieved for a general-purpose imager [26], while speeds of the order of MHz (Megahertz) have been realized using pixel-based storage for an ultra-high speed (UHS) one [27].

Apparently, certain of the novel medical imaging applications of the digital imagers can benefit from the distinguishable merits of the CMOS detectors. For example, a digital mammography detector has to be large enough for full field coverage, as well as fast enough in acquiring images, without lag and baseline drifts [21,28]. Similarly, in the industrial field, requirements include high resolution, physical ruggedness, high frame rates, cost effectiveness and portability for in-field applications. All the specifications above can be met by CMOS technology [10].

The aim of the present study was to examine the imaging performance of a state-of-the-art indirect detection CMOS X-ray detector for imaging applications, such as dual-energy (DE), in which the small pixel size could be beneficial in the detection of micro calcifications and masses, indicative of breast cancer, as well as industrial inspection applications requiring increased resolution to detect sub-millimeter structures [29]. Imaging performance metrics, such as the signal transfer property (STP), the modulation transfer function (MTF) and the normalized noise power spectrum (NNPS) were measured, to finally estimate the detective quantum efficiency (DQE) of the imaging system, following the International Electrotechnical Commission (IEC) procedures.

## 2. Materials and Methods 

The detector under investigation is the Remote RadEye HR CMOS (Teledyne DALSA, Waterloo, ON, Canada) [30] photodiode pixel array (N-well diffusion on p-type epitaxial silicon), that features an active area of 33.0 × 24.9 mm^2^, with 1650 × 1246 pixels at 20 μm pitch. The detector can be used in industrial applications, since it is small-sized and can reach tight spaces with its 12-bit USB interface. The Carestream Min-R 2190 scintillator screen (gadolinium oxysulfide activated with terbium: Gd_2_O_2_S:Tb of thickness 85 μm and screen coating weight of 33.91 mg/cm^2^) is in direct contact with the CMOS active area, while a carbon-fiber window shields from the ambient light and provides mechanical protection [30,31].

The nominal conversion gain of the detector is 155 electrons per pixel value (e^−^/PV). The nominal electronic noise (root mean square; r.m.s.) is around 310 e^−^ and the dynamic range is 66 dB. Hence, the calculated saturation charge is around 620 × 10^3^ e^−^. The maximum frame rate is 0.7 fps and the nominal average dark current is 930 e^−^/s at 23 °C. A two-meter shielded cable is included to connect the sensor head to the electronics module, where the analog video signal is processed, digitized using 12-bit ADCs and transferred to a PC [30]. The previous model of this CMOS X-ray detector was studied by Konstantinidis [32], with 60 e^−^/PV conversion gain (with a high gain option 2× available), 120 e^−^ electronic noise (r.m.s.), 72 dB dynamic range, 0.5 fps maximum frame rate, 3000 e^−^/s average dark current (at 23 °C), one meter shielded cable and 12 bit-depth ADC [32].

Due to the variety of digital imaging detector configurations (sensors and scintillators combinations) it is necessary to establish standard methods to facilitate intercomparisons between various systems, in order to juxtapose their overall performance, in terms of widely used imaging metrics, such as the MTF, NNPS and DQE [33]. To this aim, the International Electrotechnical Commission (IEC) has established a standard method (IEC 62220-1:2003 [34]; referred to as IEC 2003 for brevity) which was amended in 2015 (IEC 62220-1-1:2015 [35]; also referred to as IEC 2015). The modifications between the two protocols were examined previously in [33]. In the present work, these metrics were estimated following the 2015 version, except for the MTF that was calculated additionally according to the 2003 version, in order to compare with the MTF values provided in the manufacturer’s datasheet. The COQ plugin (Verison 2.6) for the ImageJ suite (Version 1.52a) [X]was used for the MTF, NNPS, DQE calculations [36,37].

The Del Medical Eureka radiographic system (Harisson, NY, USA) was used for the experiments. It features a rotating tungsten (W) anode with a focal spot size of 0.6 mm (“small” focal spot size selected) and an inherent filtration equivalent to 3 mm aluminum (Al). The RQA-5 radiation quality was used throughout the experiments, i.e., 70 kVp tube voltage and 6.8 mm half value layer (HVL). It was found that, in addition to the tube’s inherent filtration, another 21 mm Al (type 1100, purity 99%) must be added in order to achieve this HVL. The Al filter was placed as close to the tube as possible and the source to detector distance (SDD) was 156 cm.

The STP or X-ray sensitivity of the detector is the relationship between the mean pixel value (MPV) and Air Kerma at Detector’s surface (DAK). To obtain the DAK, the CMOS was removed altogether, and a calibrated RTI Piranha X-ray dosimeter (Mölndal, Sweden) was placed at the same position. In line with the IEC standard’s recommendations regarding the reduction of backscatter to < 0.5%, a 4 mm thick lead foil was placed at 45 cm behind the dosimeter [35]. A sequence of flat-field images was acquired at seven different exposure levels, each one consisting of five repetitions, for averaging reasons. The MPV was sampled from a 1 × 1 cm^2^ region of interest (ROI) and the system’s response curve was fitted using a linear equation of the form:

MPV = a K_a_ + b (1)

where a is the detector’s gain factor (G) [38] and b is the pixel offset at zero DAK [39].

The MTF of the detector is the variation of the output contrast as a function of the spatial frequency, and is normalized to the input contrast [40,41]. Following the IEC standard’s procedures [34,35], the MTF was measured using the slanted edge technique with the PTW Freiburg tungsten edge device, which consists of a 1 mm thick tungsten edge plate (100 × 75 mm^2^) fixed on a 3 mm thick lead plate [33,42]. The edge device was placed in contact with the detector’s entrance window at a shallow angle (1.5°–3°) with respect to the pixel rows or columns. Images were obtained at 28.1 μGy and 48.3 μGy exposures.

For the edge spread function (ESF), a 2 × 2 cm^2^ ROI was drawn, with the edge roughly at the center. The ROI’s size, smaller than the suggested by the IEC protocol, is mandated by the detector’s size. According to the IEC 62220-1:2003, the final MTF is determined by averaging the multiple MTFs obtained from the individual groups of N consecutive lines along the edge [34]. On the other hand, in the IEC 62220-1-1:2015 the final MTF is obtained by averaging the oversampled ESFs [35,43], by fitting of a modified Fermi–Dirac (F-D) distribution function of the form:

(2) $$\mathrm{Fermi}\left(x\right)=\left(\frac{c}{e^{\frac{x-a}{b}}+1}\right)+d$$

The oversampled ESFs resulted from the pixel values of the linearized data (using the inverse of the STP curve to get DAK values) of N consecutive lines across the edge. With differentiation of the fitted ESF, the line spread function (LSF) is obtained which, after the application of a Hanning filter (window width 2048 pixels) is Fourier-transformed to provide the MTF [35].

The NPS of the detector expresses the statistical variance of the output signal as a function of the spatial frequency. It was determined using flat-field images at the two exposure levels (28.1 μGy and 48.3 μGy coefficient of variation CV = 0.03%) measured with the RTI Piranha X-ray dosimeter. The normalized noise power spectrum (NNPS) tends to provide an estimation of the output noise, independent of gross exposure variations over the detector area (e.g., heel effect) [41,44]. This is achieved by fitting and then subtracting a two dimensional (2-D) second order polynomial fit S(x,y) to the original image data I(x,y) after converting to K_a_ units (see linearization process), using the inverse of the STP linear equation [35]. The average 2-D NPS is given by:

(3) $$\mathrm{NPS}\left(u,v\right)=\frac{\Delta x\Delta y}{{\mathrm{MN}}_{x}N_{y}}\overset{M}{\underset{m=1}{\sum }}{\left|\mathrm{FFT}\left\{I\left(x_{i},y_{i}\right)-S\left(x_{i},y_{i}\right)\right\}\right|}^{2}$$

where u and v denote the x and y–axis spatial frequency, respectively; Δx and Δy are the x and y—axis pixel pitches; N_x_ and N_y_ express the ROI size in the x and y axes (256 pixels according to the IEC); M is the number of ROIs used in the ensemble average; FFT denotes the fast Fourier transform operation [2].

From this, the horizontal and vertical NPS were extracted, by averaging seven rows and seven columns on each side of each axis. Axes themselves were excluded, as the IEC standard suggests, since they may contain remnant column-to-column and/or row-to-row fixed-pattern noise (FPN). These 1-D NPS were divided by the square of the averaged K_a_ [33,45], also known as large area signal, in order to obtain the NNPS in both orientations, which were then combined to obtain the radial average NNPS.

The detector’s DQE expresses its efficiency in transferring the signal to noise ratio square (SNR^2^) impinging on it, towards the output [41,46]. It is given by:

(4) $$\mathrm{DQE}\left(u\right)=\frac{{\mathrm{MTF}}^{2}\left(u\right)}{K_{a}\cdot q\cdot \mathrm{NNPS}\left(u\right)}$$

where q is the fluence per Air Kerma ratio, i.e., the number of X-ray photons per unit Air Kerma (in µGy) per mm^2^. According to the IEC 62220-1-1:2015 protocol, the value of 29653 was used for the utilized X-ray beam quality (RQA-5) [35].

## 3. Results and Discussion

**Figure 1:**
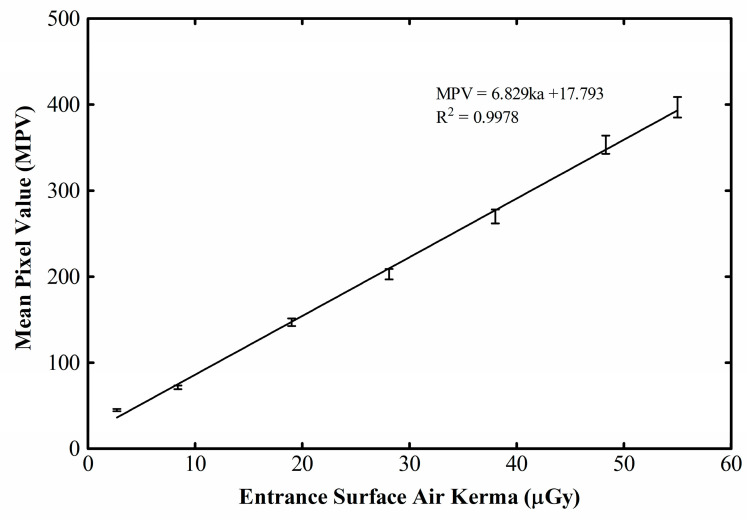
STP curve of the CMOS X-ray detector.

The MPV as a function of DAK is drawn in Figure 1 for the seven exposure levels and a linear interpolation is calculated.

The detector’s excellent linearity in the examined exposure range is obvious, since the coefficient of determination (R^2^) is 0.9978. The gain factor (G) is found 6.829 digital units (DU) per μGy. It is a higher value compared to the 5.487 measured for the previous (discontinued) version of the Remote RadEye HR detector, studied by Konstantinidis [32], at almost the same beam quality (74 kVp). It is higher as well, in comparison to the 3.829 gain at 52 kVp, of the same study [32]. In that situation, the detector was also coupled to a Gd_2_O_2_S:Tb screen of mass thickness 33.91 mg/cm^2^. The dynamic range of the present (new version) detector is practically double (up to 55 μGy, as opposed to 28 μGy of the old one), even though no loss of linearity is observed up to the exposure maxima in any case. There is also an offset of ~18 DU at zero input.

Despite the fact that two different detectors are being considered, each one with its own characteristics, increased G with increasing mean X-ray energy is an expected behavior. As described by Marshall [47] and Konstantinidis [2], with increasing mean energy of the spectrum, the number of X-ray photons per unit DAK increases as well. Every X-ray photon of higher energy causes the emission of more optical photons by the scintillator. Furthermore, higher energy X-ray photons have a greater penetration within the scintillator material, shifting the depth of interaction closer to the CMOS optical sensor and improving the detection probability of the secondary generated optical photons.

**Figure 2:**
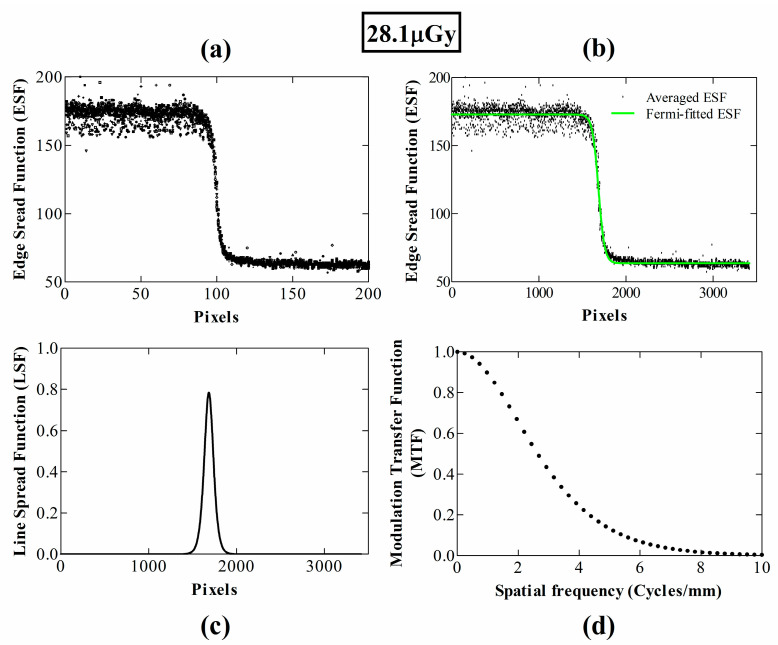
MTF calculation steps at an exposure level of 28.1 μGy, based on the IEC 2015; (**a**) all ESFs; (**b**) averaged and Fermi-fitted ESF; (**c**) LSF; (**d**) MTF.

**Figure 3:**
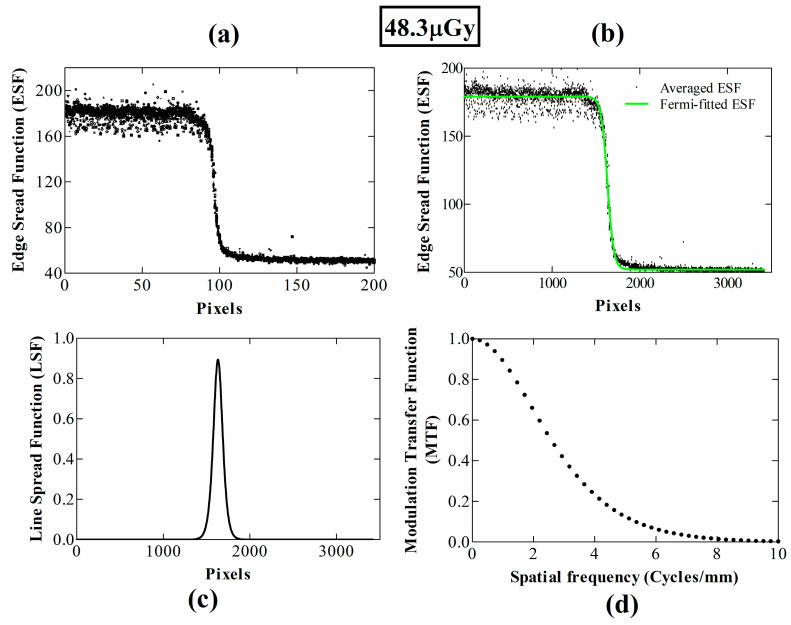
MTF calculation steps at an exposure level of 48.3 μGy, based on the IEC 2015; (**a**) all ESFs; (**b**) averaged and Fermi-fitted ESF; (**c**) LSF; (**d**) MTF.

The oversampled ESF as a function of position across the edge device (in terms of number of pixels) is depicted in Figure 2a at 28.1 μGy and in Figure 3a at 48.3 μGy. Figure 2b and Figure 3b show the corresponding averaged and Fermi-fitted ESFs. Signal fluctuations are mostly prominent in the area where the beam is not attenuated by the edge device, due to the Poisson distribution of input X-rays in spatial and temporal domains. Contrarily, in the “dark” area the fluctuations are mostly due to the electronic noise. The resulting LSFs after the fast Fourier transform (FFT) are demonstrated in Figure 2c and Figure 3c, respectively, while the MTF curves according to the IEC 62220-1-1:2015 standard are shown in Figure 2d and Figure 3d, respectively.

**Figure 4:**
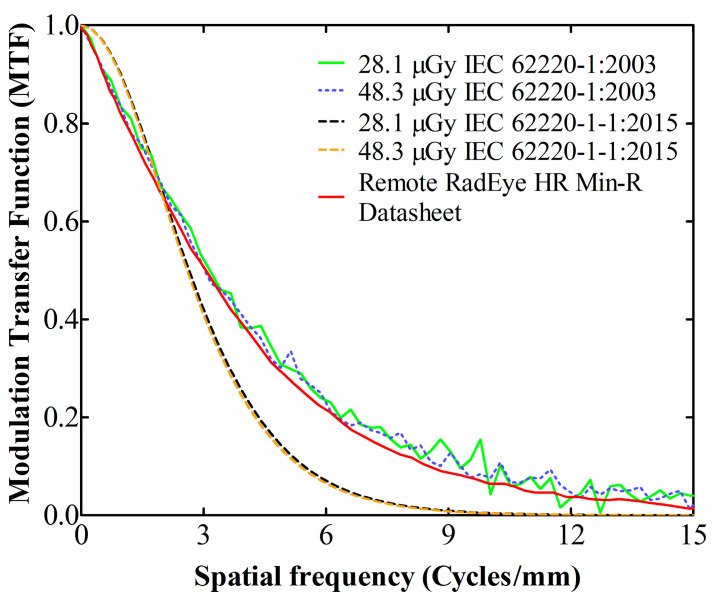
MTF comparison between measured values according to IEC 2003 and IEC 2015, along with manufacturer data [30].

For ease of comparison, these two MTF curves, along with the two MTFs according to the IEC 62220-1:2003 standard and the one extracted from the detector’s datasheet, also calculated with the 2003 protocol [30], are illustrated in Figure 4. Regarding the two IEC 62220-1-1:2015 curves, there is a complete agreement between the different exposure levels. As for the two IEC 62220-1:2003 curves, the differences are within 4%; apart from the 12% at 10 cycles/mm. Hence, regardless of the exposure value, the MTFs of the 2003 and the 2015 versions of the IEC protocol show an agreement within 10%, up to the 3 cycles/mm. After that spatial frequency they start to diverge, with the curves originating from the 2003 protocol lying higher (up to ~17% difference roughly in the range 4.5–7 cycles/mm). This difference should be attributed to the averaging method of the 2003 protocol, which influences the F-D fitting and leads to an overestimation of the MTF [33]. On the other hand, the MTF curve extracted from the detector’s datasheet follows within 6% agreement the two IEC 62220-1:2003 curves, except for the 13% difference to the 28.1 μGy curve at 10 cycles/mm. Presumably, an algorithm close to the IEC 2003 standard was used for the datasheet MTF curve calculation.

Taking the IEC 62220-1-1:2015 MTF curve as a reference, it is noted that it falls to 50% (MTF50) at 2.6 cycles/mm and to 10% (MTF10) at 5.4 cycles/mm, which marks the limiting resolution of the detector [48].

For comparison purposes, the MTF values at 50% and 10% of the examined detector [49,50] are shown in the Table 1 (1st column), along with the corresponding values of other commercial detectors, all calculated following the IEC 2003 standard. In the 2nd column, the previous (discontinued) version of the Remote RadEye HR CMOS photodiode array is shown, with an active area of 2.7 × 3.6 cm^2^, manufactured by Rad-icon Imaging Corp. (USA) with 1200 × 1600 pixels and a pixel pitch of 22.5 µm [32]. In the 3rd and 4th columns, the Dexela flat panel CMOS X-ray detector with a pixel pitch of 74.8 µm and 1944 × 1536 pixels, resulting in a 14.5 × 11.5 cm^2^ active area. It is configured with two different Cesium Iodide (CsI) scintillator screens, with thicknesses 150 and 600 μm, respectively. In the 5th column the Hamamatsu C9732DK, which is a CMOS X-ray detector with 2400 × 2400 pixels and 50 µm pixel pitch, corresponding to an active photodiode area of 12 × 12 cm^2^ [51]. Finally, in the 6th column, the Large Area Sensor (LAS) that contains 1350 × 1350 pixels at 40 µm pitch (photodiode area 5.4 × 5.4 cm^2^) [52]. As it can be seen from Table 1, the RadEye HR, in both versions, retained the highest MTF values due to the smallest pixel pitch and the gold standard 85 μm Gd_2_O_2_S:Tb screen. However, the new RadEye HR version appears to have slightly worse MTF than the previous version (4.8% and 5.5% differences at 10% and 50% of the MTF, respectively).

**Figure 5:**
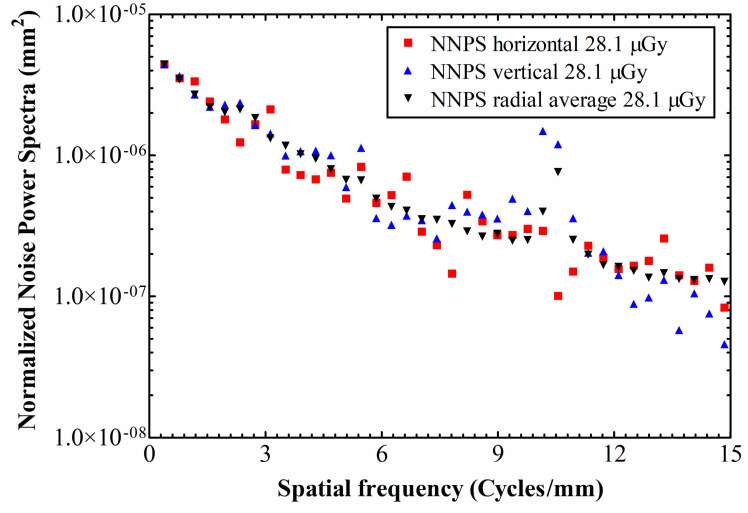
NNPS values at an exposure level of 28.1 μGy.

**Figure 6:**
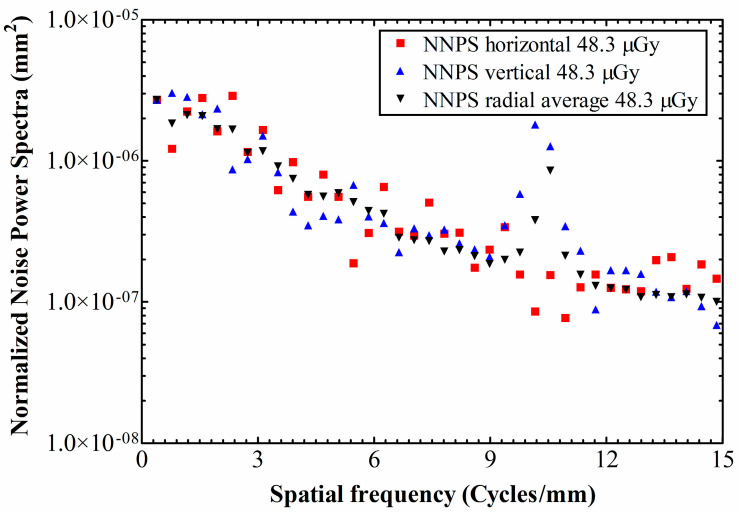
NNPS values at an exposure level of 48.3 μGy.

Horizontal and vertical 1D NNPS values, along with the radial average NNPS are depicted in Figure 5 for 28.1 μGy exposure, and in Figure 6 for 48.3 μGy. The points’ isotropy (i.e., similarity of the magnitudes between axes) for both exposures varies through the whole range of spatial frequencies; from negligible differences, up to an order of magnitude at 10.1–10.6 cycles/mm, where the vertical component exhibits a peak, probably due to remnant row-to-row FPN.

As expected, there is a tendency of NNPS decrease as the exposure increases. This is due to the fact that, with higher exposures the signal increase exceeds the noise increase (due to Poisson distribution in the detection of input X-rays). For example, at the exposure level of 28.1 the absolute NNPS value is 3.45 × 10^−6^ at 0.78 cycles/mm, whereas the corresponding value for the exposure level of 48.3 is 1.84 × 10^−6^.

**Figure 7:**
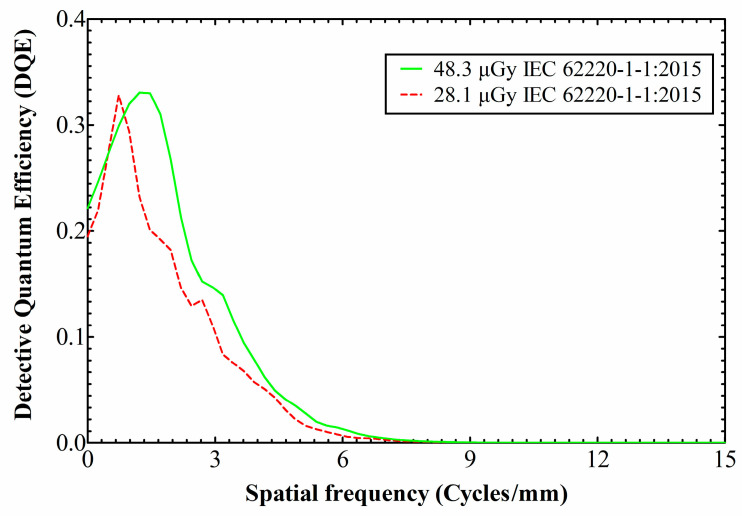
DQE curves for both the examined exposures.

In Figure 7 is drawn the DQE in both exposures of our experiments. Given that the MTF between 28.1 μGy and 48.3 μGy did not show any great differences, whereas the NNPS showed a tendency to decrease with increasing exposure, an analogous behavior is expected for the DQE curve, i.e., increased values with higher exposures. This is confirmed by the graph. Besides, the curves have similar shape, since the frequency composition is generally not affected by the exposure [53]; any shape discrepancies should be attributed to the inherent non-linearity of CMOS APS detectors, remnant FPN and electronic noise.

Other than that, the DQE peaks at ~1 cycle/mm and then drops with spatial frequency, indicating that the SNR impinging on the detector is transferred less efficiently towards its output, as the spatial frequency increases. Under ~1 cycle/mm the NNPS levels are high enough to restrain the DQE values. Subsequently, the NNPS levels fall rapidly (Figure 5 and Figure 6; logarithmic y-axes), thus letting the DQE values to build up. For the 48.3 μGy exposure, at the lowest spatial frequency limit the DQE starts from DQE(0) = 0.22, then peaks at DQE(1.3) = 0.33 and falls to 0.1 at 3.6 cycles/mm. As for the 28.1 μGy exposure, the DQE starts from DQE(0) = 0.19, then peaks at DQE(0.7) = 0.33 and falls to 0.1 at 3.0 cycles/mm.

**Figure 8:**
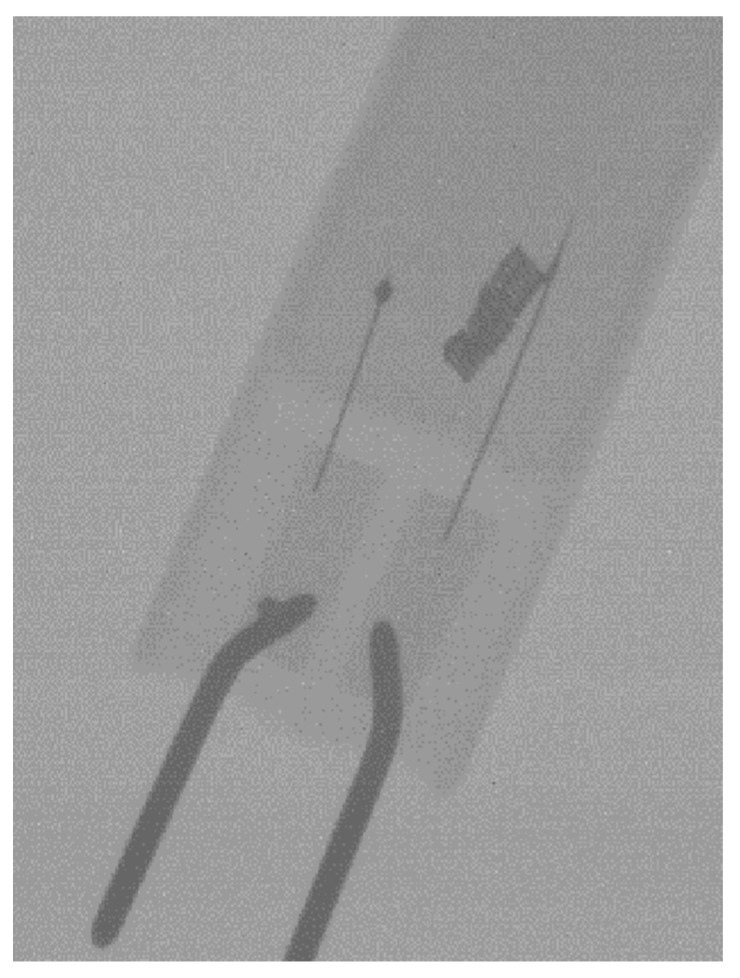
Halogen lamp radiography on the Remote RadEye HR (70 kVp 20 mAs).

As an indication of the present detector’s imaging characteristics in the NDT context, a sample radiography is displayed in Figure 8. The filament coil (0.2 mm thickness) is clearly discernible. The sample lamp (halogen type; Geyer G4 12 V 28 W, Chalkida, Greece) was placed on the detector’s surface and an exposure was taken at 70 kVp 20 mAs, using the same SDD as of the measurements, i.e., practically without geometric magnification. 

### Potential Application for Dual-Energy Imaging

The Min-R screen of the present work is made of Gd_2_O_2_S:Tb, one of the most commonly used scintillating materials coupled to digital imagers. Of its advantageous characteristics are the large effective atomic number (Z_eff_ = 60) and its density (7.3 g/cm^3^), that both favor X-ray absorption. It also exhibits a high light conversion efficiency (19%) and a high light yield (60,000 photons/MeV) [54].

In dual-energy detectors, X-rays of different energies are absorbed selectively by two scintillators of the appropriate properties, thus improving the capability to image different composition materials. DE mammography for instance, has been proven to suppress the contrast between overlapping tissues, enhancing in this way the detectability of micro-calcifications and masses [29,55,56]. In the industrial context on the other hand, dual-energy imaging can provide quantitative information of the interior structure of composite materials. Examples are flaw detection of welds or complex geometry parts, luggage inspection, food industry samples [8,9,10].

The pair ZnSe:Te and CdWO_4_ is commonly used in such arrays, with ZnSe:Te (low Z_eff_ and density) being the first layer to absorb the lower-energy photons and CdWO_4_ (high Z_eff_ and density) detecting the X-rays that traversed the first layer. Comparing the properties of Gd_2_O_2_S:Tb and CdWO_4_, one is becoming aware of the certain properties they have in common. Namely, CdWO_4_ has effective atomic number 64.2, density 7.9 g/cm^3^ and light yield 19,700 photons/MeV [57]. Its emission spectrum peaks at 495 nm, therefore it is well differentiated from that of ZnSe:Te (640 nm peak). Both spectra though, stand within the present imager’s spectral sensitivity range (0.97 matching factor with ZnSe:Te) [58,59]. These results indicate high potential for the two phosphors, Gd_2_O_2_S:Tb and ZnSe:Te, to be considered as the high- and low-energy elements, respectively, of a dual-energy flat imager.

Such a material combination has been used by Altman et al. [60] for a dual-energy computed tomography (DECT) application. Nevertheless, in this approach the sensing photodetectors are placed next to every row of phosphor pair, i.e., the light detection takes place through the scintillators’ side surfaces. This means an insensitive area of 0.125 mm thickness next to each phosphor row, as well as the necessity to radiation-shield these photodetectors [60].

The previous version of the examined detector, also having Gd_2_O_2_S:Tb phosphor, has been used in dual-energy breast imaging resulting in the detectability of 150 μm thick calcifications. The small pixel pitch allowed post processing in the final images and thus a 93 μm thick calcification was visible [55]. Considering that systems used in digital mammography can detect calcifications as small as 130 μm [61], such a detector has the potential to be used in dual-energy imaging. The same detector was also used in a study for characterization between malignant and benign calcifications. Based on the results of the study, such characterization could be accomplished for calcification thicknesses of 300 μm or higher [29]. Although compared to the previous version of RadEye HR the new version somehow has lower resolution (4.8% and 5.5% differences at 10% and 50% of the MTF, respectively; please see Table 1), such a slight reduction cannot substantially affect its diagnostic performance.

In conclusion, for those applications requesting low to medium diagnostic X-ray energies, the scintillator pair Gd_2_O_2_S:Tb and ZnSe:Te with the present CMOS imager could be considered as a possible DE array. Diverse approaches have been proposed, yet none of which utilizing these materials in a 2-D panel configuration, to the best of our knowledge. One could be similar to the dual-energy dual-color approach of Maier et al. [62]; the two phosphors are layered together, and the signal is differentiated by means of their different emission wavelengths and an optical layout consisting of a dichroic mirror and lenses. Another could be analogous to the Han et al. [63] solution of a sandwich detector; two Gd_2_O_2_S:Tb phosphors of different thicknesses are separated by an optically-opaque, radio-translucent foil and a CMOS is coupled on each side.

## 4. Conclusions

The present NDT CMOS-based detector exhibits excellent linearity across the examined exposure range and a higher gain factor compared to the older 22.5 μm CMOS. The MTF calculated according to the IEC 2003 standard is in close agreement with the CMOS manufacturer’s MTF curve, whereas the MTF calculated with IEC 2015 is lower in the higher frequency range, due to the averaging method of IEC 2003 that leads to an overestimation of the MTF. MTF values are higher compared to other commercial CMOS detectors. However, the previous, discontinued, version of the detector under investigation performs slightly better in terms of the MTF, despite its coarser pixel pitch (22.5 μm instead of 20.0 μm). Regarding the detector’s phosphor material, Gd_2_O_2_S:Tb, and based on its properties’ pertinence to those of CdWO_4_, dual-energy application is discussed. Specifically, the present CMOS with its Gd_2_O_2_S:Tb scintillator, in conjunction with ZnSe: Te, appears as a promising candidate for a dual-energy flat imager aimed at the NDT and medical fields.

## Figures and Tables

**Table 1:** MTF values at 10% and 50% for various CMOS X-ray detectors [32].

Detector	RadEye HR 20.0 μm Pitch	RadEye HR 22.5 μm Pitch	Dexela 150 μm CsI	Dexela 600 μm CsI	Hamamatsu C9732DK	LAS
Pixel Pitch (μm)	20.0	22.5	74.8	74.8	50	40
Dimensions (pixels)	1650 × 1246	1200 × 1600	1944 × 1536	1944 × 1536	2400 × 2400	1350 × 1350
Dimensions (cm^2^)	2.49 × 3.3	2.7 × 3.6	14.5 × 11.5	14.5 × 11.5	12 × 12	5.4 × 5.4
MTF@10% (cycles/mm)	10.4	10.9	7.7	4.4	9	4.1
MTF@50% (cycles/mm)	3.6	3.8	2.7	1.3	2.7	1.3

## Data Availability

Data is contained within the article..
